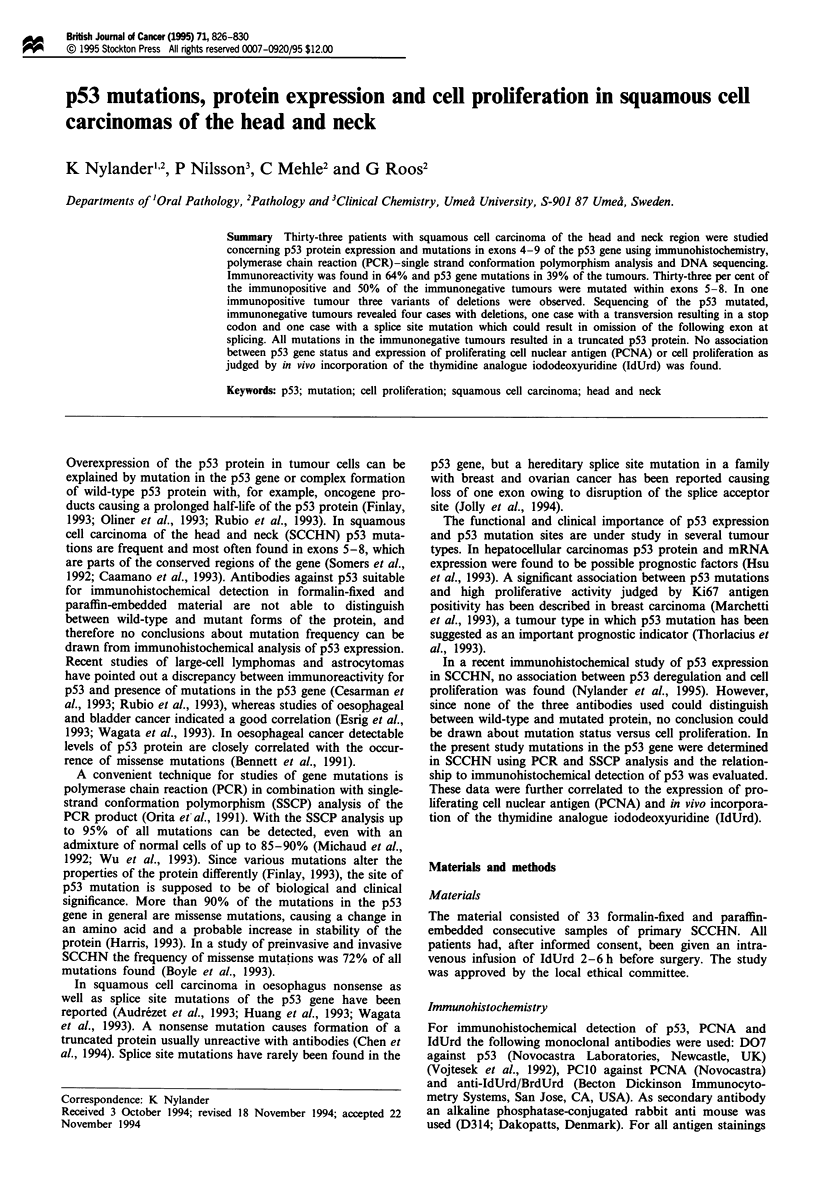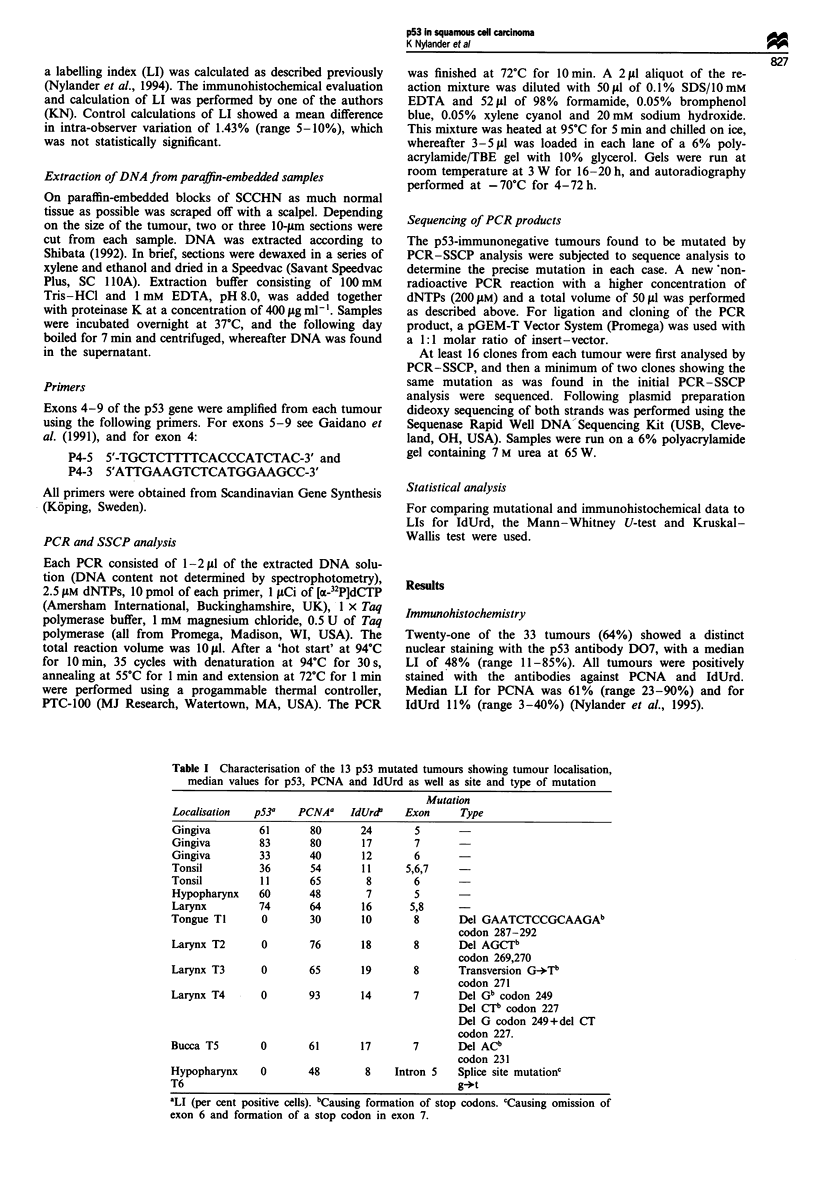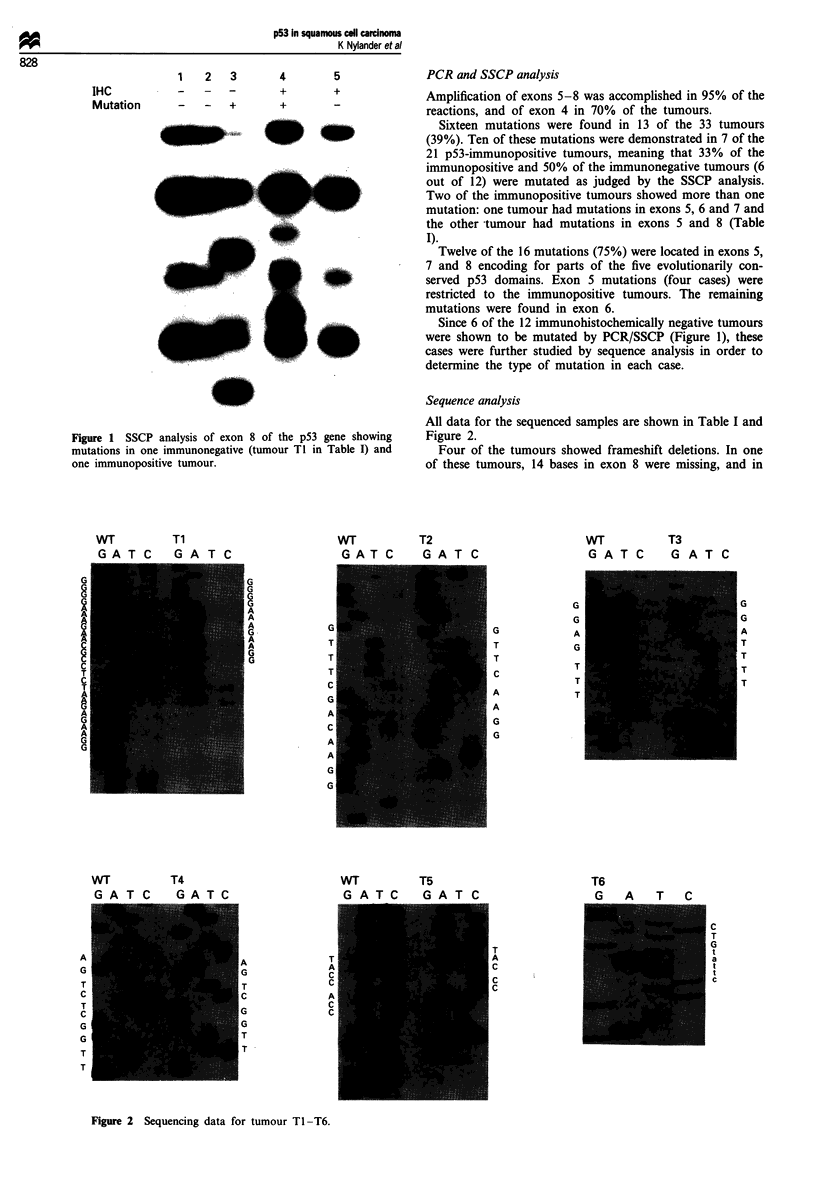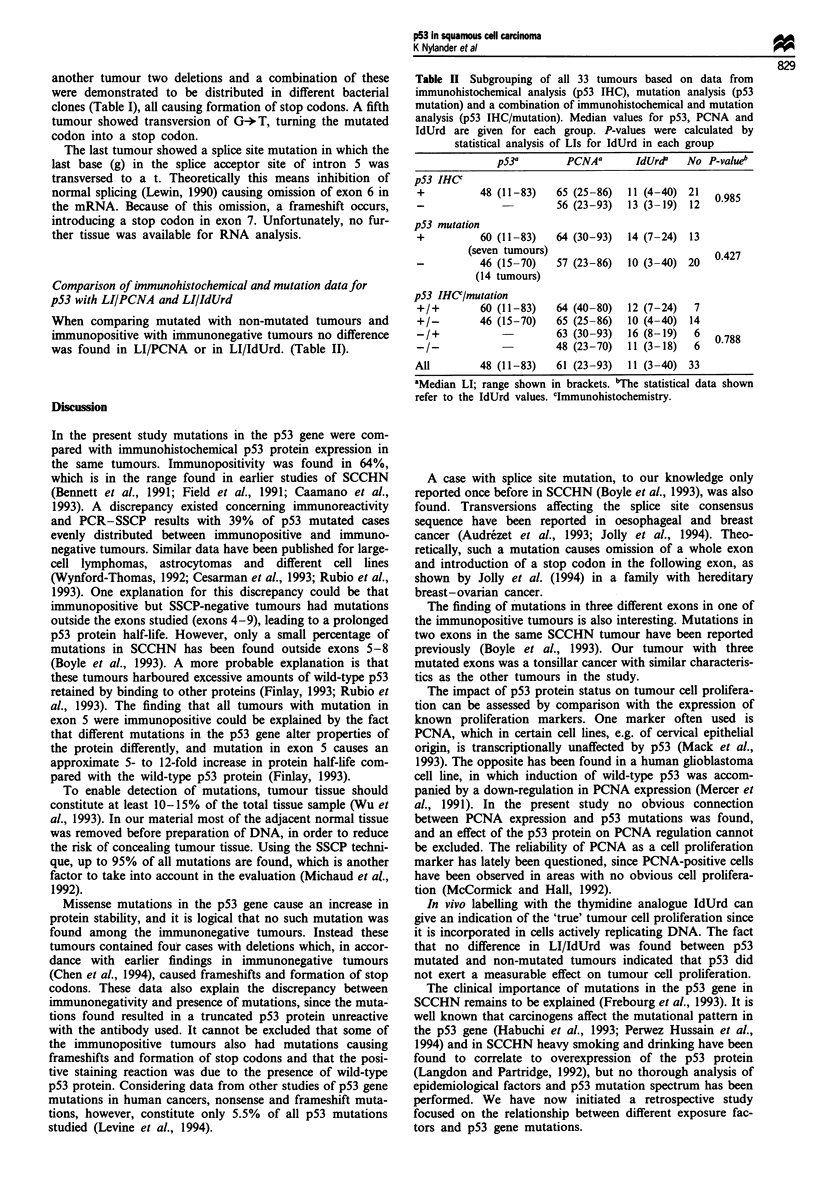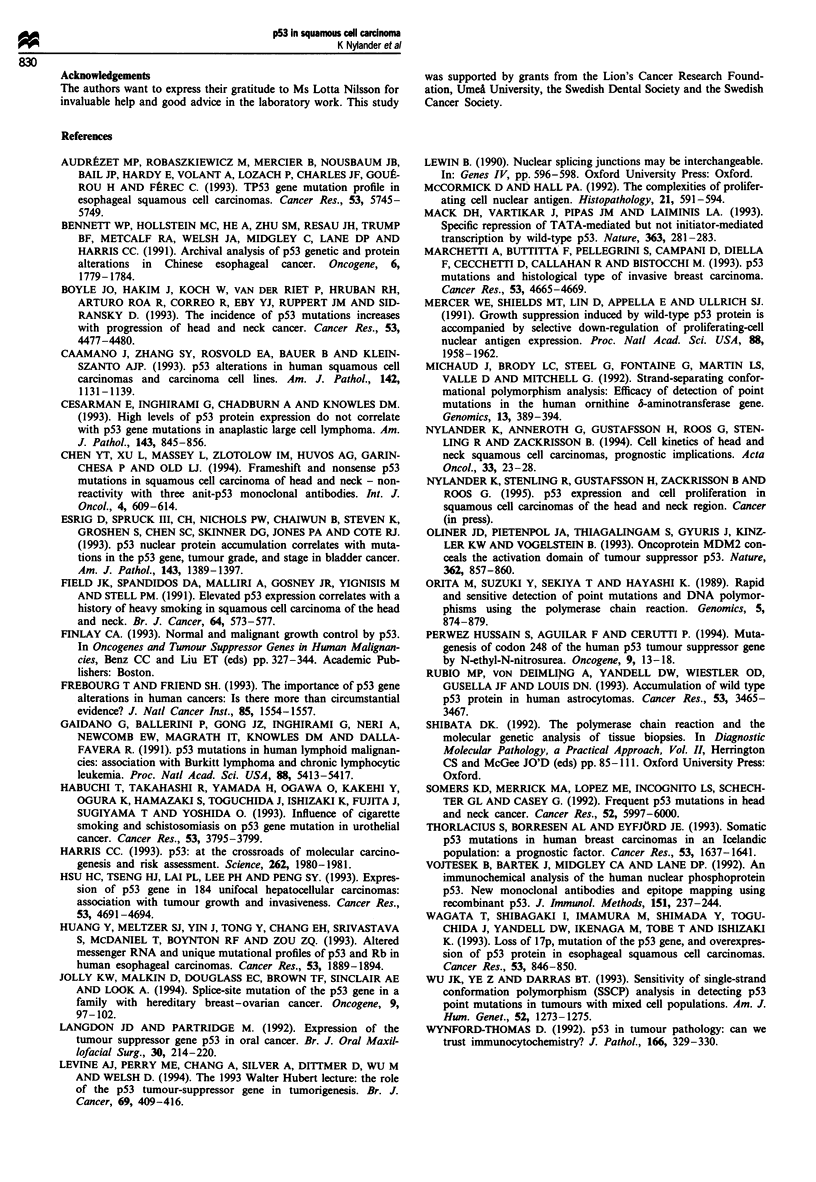# p53 mutations, protein expression and cell proliferation in squamous cell carcinomas of the head and neck.

**DOI:** 10.1038/bjc.1995.159

**Published:** 1995-04

**Authors:** K. Nylander, P. Nilsson, C. Mehle, G. Roos

**Affiliations:** Department of Oral Pathology, Umeå University, Sweden.

## Abstract

**Images:**


British Journal of Cancer (1995) 71, 826-830
© 1995 Stockton Press All rights reserved 0007-0920/95 $12.00

### p53 mutations, protein expression and cell proliferation in squamous cell carcinomas of the head and neck

K Nylander[1,2], P Nilsson[3], C Mehle[2] and G Roos[2]

*Departments of [1]Oral Pathology, [2]Pathology and [3]Clinical Chemistry, Umeå University, S-901 87 Umeå, Sweden.*

**Summary** Thirty-three patients with squamous cell carcinoma of the head and neck region were studied concerning p53 protein expression and mutations in exons 4-9 of the p53 gene using immunohistochemistry, polymerase chain reaction (PCR)-single strand conformation polymorphism analysis and DNA sequencing. Immunoreactivity was found in 64% and p53 gene mutations in 39% of the tumours. Thirty-three per cent of the immunopositive and 50% of the immunonegative tumours were mutated within exons 5-8. In one immunopositive tumour three variants of deletions were observed. Sequencing of the p53 mutated, immunonegative tumours revealed four cases with deletions, one case with a transversion resulting in a stop codon and one case with a splice site mutation which could result in omission of the following exon at splicing. All mutations in the immunonegative tumours resulted in a truncated p53 protein. No association between p53 gene status and expression of proliferating cell nuclear antigen (PCNA) or cell proliferation as judged by *in vivo* incorporation of the thymidine analogue iododeoxyuridine (IdUrd) was found.

**Keywords:** p53; mutation; cell proliferation; squamous cell carcinoma; head and neck

Overexpression of the p53 protein in tumour cells can be explained by mutation in the p53 gene or complex formation of wild-type p53 protein with, for example, oncogene products causing a prolonged half-life of the p53 protein (Finlay, 1993; Oliner *et al.*, 1993; Rubio *et al.*, 1993). In squamous cell carcinoma of the head and neck (SCCHN) p53 mutations are frequent and most often found in exons 5-8, which are parts of the conserved regions of the gene (Somers *et al.*, 1992; Caamano *et al.*, 1993). Antibodies against p53 suitable for immunohistochemical detection in formalin-fixed and paraffin-embedded material are not able to distinguish between wild-type and mutant forms of the protein, and therefore no conclusions about mutation frequency can be drawn from immunohistochemical analysis of p53 expression. Recent studies of large-cell lymphomas and astrocytomas have pointed out a discrepancy between immunoreactivity for p53 and presence of mutations in the p53 gene (Cesarman *et al.*, 1993; Rubio *et al.*, 1993), whereas studies of oesophageal and bladder cancer indicated a good correlation (Esrig *et al.*, 1993; Wagata *et al.*, 1993). In oesophageal cancer detectable levels of p53 protein are closely correlated with the occurrence of missense mutations (Bennett *et al.*, 1991).

A convenient technique for studies of gene mutations is polymerase chain reaction (PCR) in combination with single-strand conformation polymorphism (SSCP) analysis of the PCR product (Orita *et al.*, 1991). With the SSCP analysis up to 95% of all mutations can be detected, even with an admixture of normal cells of up to 85-90% (Michaud *et al.*, 1992; Wu *et al.*, 1993). Since various mutations alter the properties of the protein differently (Finlay, 1993), the site of p53 mutation is supposed to be of biological and clinical significance. More than 90% of the mutations in the p53 gene in general are missense mutations, causing a change in an amino acid and a probable increase in stability of the protein (Harris, 1993). In a study of preinvasive and invasive SCCHN the frequency of missense mutations was 72% of all mutations found (Boyle *et al.*, 1993).

In squamous cell carcinoma in oesophagus nonsense as well as splice site mutations of the p53 gene have been reported (Audrézet *et al.*, 1993; Huang *et al.*, 1993; Wagata *et al.*, 1993). A nonsense mutation causes formation of a truncated protein usually unreactive with antibodies (Chen *et al.*, 1994). Splice site mutations have rarely been found in the p53 gene, but a hereditary splice site mutation in a family with breast and ovarian cancer has been reported causing loss of one exon owing to disruption of the splice acceptor site (Jolly *et al.*, 1994).

The functional and clinical importance of p53 expression and p53 mutation sites are under study in several tumour types. In hepatocellular carcinomas p53 protein and mRNA expression were found to be possible prognostic factors (Hsu *et al.*, 1993). A significant association between p53 mutations and high proliferative activity judged by Ki67 antigen positivity has been described in breast carcinoma (Marchetti *et al.*, 1993), a tumour type in which p53 mutation has been suggested as an important prognostic indicator (Thorlacius *et al.*, 1993).

In a recent immunohistochemical study of p53 expression in SCCHN, no association between p53 deregulation and cell proliferation was found (Nylander *et al.*, 1995). However, since none of the three antibodies used could distinguish between wild-type and mutated protein, no conclusion could be drawn about mutation status versus cell proliferation. In the present study mutations in the p53 gene were determined in SCCHN using PCR and SSCP analysis and the relationship to immunohistochemical detection of p53 was evaluated. These data were further correlated to the expression of proliferating cell nuclear antigen (PCNA) and *in vivo* incorporation of the thymidine analogue iododeoxyuridine (IdUrd).

### Materials and methods

### *Materials*

The material consisted of 33 formalin-fixed and paraffin-embedded consecutive samples of primary SCCHN. All patients had, after informed consent, been given an intravenous infusion of IdUrd 2-6 h before surgery. The study was approved by the local ethical committee.

### *Immunohistochemistry*

For immunohistochemical detection of p53, PCNA and IdUrd the following monoclonal antibodies were used: DO7 against p53 (Novocastra Laboratories, Newcastle, UK) (Vojtesek *et al.*, 1992), PC10 against PCNA (Novocastra) and anti-IdUrd/BrdUrd (Becton Dickinson Immunocytometry Systems, San Jose, CA, USA). As secondary antibody an alkaline phosphatase-conjugated rabbit anti mouse was used (D314; Dakopatts, Denmark). For all antigen stainings

Correspondence: K Nylander
Received 3 October 1994; revised 18 November 1994; accepted 22 November 1994

a labelling index (LI) was calculated as described previously (Nylander *et al.*, 1994). The immunohistochemical evaluation and calculation of LI was performed by one of the authors (KN). Control calculations of LI showed a mean difference in intra-observer variation of 1.43% (range 5–10%), which was not statistically significant.

### *Extraction of DNA from paraffin-embedded samples*

On paraffin-embedded blocks of SCCHN as much normal tissue as possible was scraped off with a scalpel. Depending on the size of the tumour, two or three 10-μm sections were cut from each sample. DNA was extracted according to Shibata (1992). In brief, sections were dewaxed in a series of xylene and ethanol and dried in a Speedvac (Savant Speedvac Plus, SC 110A). Extraction buffer consisting of 100 mM Tris-HCl and 1 mM EDTA, pH 8.0, was added together with proteinase K at a concentration of 400 μg $ml^{-1}$. Samples were incubated overnight at 37°C, and the following day boiled for 7 min and centrifuged, whereafter DNA was found in the supernatant.

### *Primers*

Exons 4–9 of the p53 gene were amplified from each tumour using the following primers. For exons 5–9 see Gaidano *et al.* (1991), and for exon 4:

P4-5 5′-TGCTCTTTTCACCCATCTAC-3′ and
P4-3 5′ATTGAAGTCTCATGGAAGCC-3′

All primers were obtained from Scandinavian Gene Synthesis (Köping, Sweden).

### *PCR and SSCP analysis*

Each PCR consisted of 1–2 μl of the extracted DNA solution (DNA content not determined by spectrophotometry), 2.5 μM dNTPs, 10 pmol of each primer, 1 μCi of [α-$^{32}$P]dCTP (Amersham International, Buckinghamshire, UK), 1 × *Taq* polymerase buffer, 1 mM magnesium chloride, 0.5 U of *Taq* polymerase (all from Promega, Madison, WI, USA). The total reaction volume was 10 μl. After a 'hot start' at 94°C for 10 min, 35 cycles with denaturation at 94°C for 30 s, annealing at 55°C for 1 min and extension at 72°C for 1 min were performed using a progammable thermal controller, PTC-100 (MJ Research, Watertown, MA, USA). The PCR was finished at 72°C for 10 min. A 2 μl aliquot of the reaction mixture was diluted with 50 μl of 0.1% SDS/10 mM EDTA and 52 μl of 98% formamide, 0.05% bromphenol blue, 0.05% xylene cyanol and 20 mM sodium hydroxide. This mixture was heated at 95°C for 5 min and chilled on ice, whereafter 3–5 μl was loaded in each lane of a 6% polyacrylamide/TBE gel with 10% glycerol. Gels were run at room temperature at 3 W for 16–20 h, and autoradiography performed at −70°C for 4–72 h.

### *Sequencing of PCR products*

The p53-immunonegative tumours found to be mutated by PCR–SSCP analysis were subjected to sequence analysis to determine the precise mutation in each case. A new non-radioactive PCR reaction with a higher concentration of dNTPs (200 μM) and a total volume of 50 μl was performed as described above. For ligation and cloning of the PCR product, a pGEM-T Vector System (Promega) was used with a 1:1 molar ratio of insert–vector.

At least 16 clones from each tumour were first analysed by PCR–SSCP, and then a minimum of two clones showing the same mutation as was found in the initial PCR–SSCP analysis were sequenced. Following plasmid preparation dideoxy sequencing of both strands was performed using the Sequenase Rapid Well DNA Sequencing Kit (USB, Cleveland, OH, USA). Samples were run on a 6% polyacrylamide gel containing 7 M urea at 65 W.

### *Statistical analysis*

For comparing mutational and immunohistochemical data to LIs for IdUrd, the Mann–Whitney *U*-test and Kruskal–Wallis test were used.

### Results

### *Immunohistochemistry*

Twenty-one of the 33 tumours (64%) showed a distinct nuclear staining with the p53 antibody DO7, with a median LI of 48% (range 11–85%). All tumours were positively stained with the antibodies against PCNA and IdUrd. Median LI for PCNA was 61% (range 23–90%) and for IdUrd 11% (range 3–40%) (Nylander *et al.*, 1995).

**Table I** Characterisation of the 13 p53 mutated tumours showing tumour localisation, median values for p53, PCNA and IdUrd as well as site and type of mutation

| *Localisation* | *p53*[a] | *PCNA*[a] | *IdUrd*[a] | *Mutation* *Exon* | *Type* |
|---|---|---|---|---|---|
| Gingiva | 61 | 80 | 24 | 5 | — |
| Gingiva | 83 | 80 | 17 | 7 | — |
| Gingiva | 33 | 40 | 12 | 6 | — |
| Tonsil | 36 | 54 | 11 | 5,6,7 | — |
| Tonsil | 11 | 65 | 8 | 6 | — |
| Hypopharynx | 60 | 48 | 7 | 5 | — |
| Larynx | 74 | 64 | 16 | 5,8 | — |
| Tongue T1 | 0 | 30 | 10 | 8 | Del GAATCTCCGCAAGA[b] codon 287–292 |
| Larynx T2 | 0 | 76 | 18 | 8 | Del AGCT[b] codon 269,270 |
| Larynx T3 | 0 | 65 | 19 | 8 | Transversion G→T[b] codon 271 |
| Larynx T4 | 0 | 93 | 14 | 7 | Del G[b] codon 249 Del CT[b] codon 227 Del G codon 249+del CT codon 227. |
| Bucca T5 | 0 | 61 | 17 | 7 | Del AC[b] codon 231 |
| Hypopharynx T6 | 0 | 48 | 8 | Intron 5 | Splice site mutation[c] g→t |

[a]LI (per cent positive cells). [b]Causing formation of stop codons. [c]Causing omission of exon 6 and formation of a stop codon in exon 7.

**Figure 1** SSCP analysis of exon 8 of the p53 gene showing mutations in one immunonegative (tumour T1 in Table I) and one immunopositive tumour.

*PCR and SSCP analysis*

Amplification of exons 5–8 was accomplished in 95% of the reactions, and of exon 4 in 70% of the tumours.

Sixteen mutations were found in 13 of the 33 tumours (39%). Ten of these mutations were demonstrated in 7 of the 21 p53-immunopositive tumours, meaning that 33% of the immunopositive and 50% of the immunonegative tumours (6 out of 12) were mutated as judged by the SSCP analysis. Two of the immunopositive tumours showed more than one mutation: one tumour had mutations in exons 5, 6 and 7 and the other tumour had mutations in exons 5 and 8 (Table I).

Twelve of the 16 mutations (75%) were located in exons 5, 7 and 8 encoding for parts of the five evolutionarily conserved p53 domains. Exon 5 mutations (four cases) were restricted to the immunopositive tumours. The remaining mutations were found in exon 6.

Since 6 of the 12 immunohistochemically negative tumours were shown to be mutated by PCR/SSCP (Figure 1), these cases were further studied by sequence analysis in order to determine the type of mutation in each case.

*Sequence analysis*

All data for the sequenced samples are shown in Table I and Figure 2.

Four of the tumours showed frameshift deletions. In one of these tumours, 14 bases in exon 8 were missing, and in

**Figure 2** Sequencing data for tumour T1–T6.

another tumour two deletions and a combination of these were demonstrated to be distributed in different bacterial clones (Table I), all causing formation of stop codons. A fifth tumour showed transversion of G→T, turning the mutated codon into a stop codon.

The last tumour showed a splice site mutation in which the last base (g) in the splice acceptor site of intron 5 was transversed to a t. Theoretically this means inhibition of normal splicing (Lewin, 1990) causing omission of exon 6 in the mRNA. Because of this omission, a frameshift occurs, introducing a stop codon in exon 7. Unfortunately, no further tissue was available for RNA analysis.

*Comparison of immunohistochemical and mutation data for p53 with LI/PCNA and LI/IdUrd*

When comparing mutated with non-mutated tumours and immunopositive with immunonegative tumours no difference was found in LI/PCNA or in LI/IdUrd. (Table II).

**Table II** Subgrouping of all 33 tumours based on data from immunohistochemical analysis (p53 IHC), mutation analysis (p53 mutation) and a combination of immunohistochemical and mutation analysis (p53 IHC/mutation). Median values for p53, PCNA and IdUrd are given for each group. *P*-values were calculated by statistical analysis of LIs for IdUrd in each group

| | *p53*[a] | *PCNA*[a] | *IdUrd*[a] | *No* | *P-value*[b] |
|---|---|---|---|---|---|
| *p53 IHC*[c] | | | | | |
| + | 48 (11–83) | 65 (25–86) | 11 (4–40) | 21 | 0.985 |
| – | — | 56 (23–93) | 13 (3–19) | 12 | |
| *p53 mutation* | | | | | |
| + | 60 (11–83) (seven tumours) | 64 (30–93) | 14 (7–24) | 13 | 0.427 |
| – | 46 (15–70) (14 tumours) | 57 (23–86) | 10 (3–40) | 20 | |
| *p53 IHC*[c]*/mutation* | | | | | |
| +/+ | 60 (11–83) | 64 (40–80) | 12 (7–24) | 7 | 0.788 |
| +/– | 46 (15–70) | 65 (25–86) | 10 (4–40) | 14 | |
| –/+ | — | 63 (30–93) | 16 (8–19) | 6 | |
| –/– | — | 48 (23–70) | 11 (3–18) | 6 | |
| All | 48 (11–83) | 61 (23–93) | 11 (3–40) | 33 | |

[a]Median LI; range shown in brackets. [b]The statistical data shown refer to the IdUrd values. [c]Immunohistochemistry.

### Discussion

In the present study mutations in the p53 gene were compared with immunohistochemical p53 protein expression in the same tumours. Immunopositivity was found in 64%, which is in the range found in earlier studies of SCCHN (Bennett *et al.*, 1991; Field *et al.*, 1991; Caamano *et al.*, 1993). A discrepancy existed concerning immunoreactivity and PCR–SSCP results with 39% of p53 mutated cases evenly distributed between immunopositive and immunonegative tumours. Similar data have been published for large-cell lymphomas, astrocytomas and different cell lines (Wynford-Thomas, 1992; Cesarman *et al.*, 1993; Rubio *et al.*, 1993). One explanation for this discrepancy could be that immunopositive but SSCP-negative tumours had mutations outside the exons studied (exons 4–9), leading to a prolonged p53 protein half-life. However, only a small percentage of mutations in SCCHN has been found outside exons 5–8 (Boyle *et al.*, 1993). A more probable explanation is that these tumours harboured excessive amounts of wild-type p53 retained by binding to other proteins (Finlay, 1993; Rubio *et al.*, 1993). The finding that all tumours with mutation in exon 5 were immunopositive could be explained by the fact that different mutations in the p53 gene alter properties of the protein differently, and mutation in exon 5 causes an approximate 5- to 12-fold increase in protein half-life compared with the wild-type p53 protein (Finlay, 1993).

To enable detection of mutations, tumour tissue should constitute at least 10–15% of the total tissue sample (Wu *et al.*, 1993). In our material most of the adjacent normal tissue was removed before preparation of DNA, in order to reduce the risk of concealing tumour tissue. Using the SSCP technique, up to 95% of all mutations are found, which is another factor to take into account in the evaluation (Michaud *et al.*, 1992).

Missense mutations in the p53 gene cause an increase in protein stability, and it is logical that no such mutation was found among the immunonegative tumours. Instead these tumours contained four cases with deletions which, in accordance with earlier findings in immunonegative tumours (Chen *et al.*, 1994), caused frameshifts and formation of stop codons. These data also explain the discrepancy between immunonegativity and presence of mutations, since the mutations found resulted in a truncated p53 protein unreactive with the antibody used. It cannot be excluded that some of the immunopositive tumours also had mutations causing frameshifts and formation of stop codons and that the positive staining reaction was due to the presence of wild-type p53 protein. Considering data from other studies of p53 gene mutations in human cancers, nonsense and frameshift mutations, however, constitute only 5.5% of all p53 mutations studied (Levine *et al.*, 1994).

A case with splice site mutation, to our knowledge only reported once before in SCCHN (Boyle *et al.*, 1993), was also found. Transversions affecting the splice site consensus sequence have been reported in oesophageal and breast cancer (Audrézet *et al.*, 1993; Jolly *et al.*, 1994). Theoretically, such a mutation causes omission of a whole exon and introduction of a stop codon in the following exon, as shown by Jolly *et al.* (1994) in a family with hereditary breast–ovarian cancer.

The finding of mutations in three different exons in one of the immunopositive tumours is also interesting. Mutations in two exons in the same SCCHN tumour have been reported previously (Boyle *et al.*, 1993). Our tumour with three mutated exons was a tonsillar cancer with similar characteristics as the other tumours in the study.

The impact of p53 protein status on tumour cell proliferation can be assessed by comparison with the expression of known proliferation markers. One marker often used is PCNA, which in certain cell lines, e.g. of cervical epithelial origin, is transcriptionally unaffected by p53 (Mack *et al.*, 1993). The opposite has been found in a human glioblastoma cell line, in which induction of wild-type p53 was accompanied by a down-regulation in PCNA expression (Mercer *et al.*, 1991). In the present study no obvious connection between PCNA expression and p53 mutations was found, and an effect of the p53 protein on PCNA regulation cannot be excluded. The reliability of PCNA as a cell proliferation marker has lately been questioned, since PCNA-positive cells have been observed in areas with no obvious cell proliferation (McCormick and Hall, 1992).

*In vivo* labelling with the thymidine analogue IdUrd can give an indication of the 'true' tumour cell proliferation since it is incorporated in cells actively replicating DNA. The fact that no difference in LI/IdUrd was found between p53 mutated and non-mutated tumours indicated that p53 did not exert a measurable effect on tumour cell proliferation.

The clinical importance of mutations in the p53 gene in SCCHN remains to be explained (Frebourg *et al.*, 1993). It is well known that carcinogens affect the mutational pattern in the p53 gene (Habuchi *et al.*, 1993; Perwez Hussain *et al.*, 1994) and in SCCHN heavy smoking and drinking have been found to correlate to overexpression of the p53 protein (Langdon and Partridge, 1992), but no thorough analysis of epidemiological factors and p53 mutation spectrum has been performed. We have now initiated a retrospective study focused on the relationship between different exposure factors and p53 gene mutations.

**Acknowledgements**
The authors want to express their gratitude to Ms Lotta Nilsson for invaluable help and good advice in the laboratory work. This study was supported by grants from the Lion's Cancer Research Foundation, Umeå University, the Swedish Dental Society and the Swedish Cancer Society.

### References

AUDRÉZET MP, ROBASZKIEWICZ M, MERCIER B, NOUSBAUM JB, BAIL JP, HARDY E, VOLANT A, LOZACH P, CHARLES JF, GOUÉROU H AND FÉREC C. (1993). TP53 gene mutation profile in esophageal squamous cell carcinomas. *Cancer Res.*, **53**, 5745–5749.

BENNETT WP, HOLLSTEIN MC, HE A, ZHU SM, RESAU JH, TRUMP BF, METCALF RA, WELSH JA, MIDGLEY C, LANE DP AND HARRIS CC. (1991). Archival analysis of p53 genetic and protein alterations in Chinese esophageal cancer. *Oncogene*, **6**, 1779–1784.

BOYLE JO, HAKIM J, KOCH W, VAN DER RIET P, HRUBAN RH, ARTURO ROA R, CORREO R, EBY YJ, RUPPERT JM AND SIDRANSKY D. (1993). The incidence of p53 mutations increases with progression of head and neck cancer. *Cancer Res.*, **53**, 4477–4480.

CAAMANO J, ZHANG SY, ROSVOLD EA, BAUER B AND KLEIN-SZANTO AJP. (1993). p53 alterations in human squamous cell carcinomas and carcinoma cell lines. *Am. J. Pathol.*, **142**, 1131–1139.

CESARMAN E, INGHIRAMI G, CHADBURN A AND KNOWLES DM. (1993). High levels of p53 protein expression do not correlate with p53 gene mutations in anaplastic large cell lymphoma. *Am. J. Pathol.*, **143**, 845–856.

CHEN YT, XU L, MASSEY L, ZLOTOLOW IM, HUVOS AG, GARIN-CHESA P AND OLD LJ. (1994). Frameshift and nonsense p53 mutations in squamous cell carcinoma of head and neck – non-reactivity with three anit-p53 monoclonal antibodies. *Int. J. Oncol.*, **4**, 609–614.

ESRIG D, SPRUCK III, CH, NICHOLS PW, CHAIWUN B, STEVEN K, GROSHEN S, CHEN SC, SKINNER DG, JONES PA AND COTE RJ. (1993). p53 nuclear protein accumulation correlates with mutations in the p53 gene, tumour grade, and stage in bladder cancer. *Am. J. Pathol.*, **143**, 1389–1397.

FIELD JK, SPANDIDOS DA, MALLIRI A, GOSNEY JR, YIGNISIS M AND STELL PM. (1991). Elevated p53 expression correlates with a history of heavy smoking in squamous cell carcinoma of the head and neck. *Br. J. Cancer*, **64**, 573–577.

FINLAY CA. (1993). Normal and malignant growth control by p53. In *Oncogenes and Tumour Suppressor Genes in Human Malignancies*, Benz CC and Liu ET (eds) pp. 327–344. Academic Publishers: Boston.

FREBOURG T AND FRIEND SH. (1993). The importance of p53 gene alterations in human cancers: Is there more than circumstantial evidence? *J. Natl Cancer Inst.*, **85**, 1554–1557.

GAIDANO G, BALLERINI P, GONG JZ, INGHIRAMI G, NERI A, NEWCOMB EW, MAGRATH IT, KNOWLES DM AND DALLA-FAVERA R. (1991). p53 mutations in human lymphoid malignancies: association with Burkitt lymphoma and chronic lymphocytic leukemia. *Proc. Natl Acad. Sci. USA*, **88**, 5413–5417.

HABUCHI T, TAKAHASHI R, YAMADA H, OGAWA O, KAKEHI Y, OGURA K, HAMAZAKI S, TOGUCHIDA J, ISHIZAKI K, FUJITA J, SUGIYAMA T AND YOSHIDA O. (1993). Influence of cigarette smoking and schistosomiasis on p53 gene mutation in urothelial cancer. *Cancer Res.*, **53**, 3795–3799.

HARRIS CC. (1993). p53: at the crossroads of molecular carcinogenesis and risk assessment. *Science*, **262**, 1980–1981.

HSU HC, TSENG HJ, LAI PL, LEE PH AND PENG SY. (1993). Expression of p53 gene in 184 unifocal hepatocellular carcinomas: association with tumour growth and invasiveness. *Cancer Res.*, **53**, 4691–4694.

HUANG Y, MELTZER SJ, YIN J, TONG Y, CHANG EH, SRIVASTAVA S, McDANIEL T, BOYNTON RF AND ZOU ZQ. (1993). Altered messenger RNA and unique mutational profiles of p53 and Rb in human esophageal carcinomas. *Cancer Res.*, **53**, 1889–1894.

JOLLY KW, MALKIN D, DOUGLASS EC, BROWN TF, SINCLAIR AE AND LOOK A. (1994). Splice-site mutation of the p53 gene in a family with hereditary breast-ovarian cancer. *Oncogene*, **9**, 97–102.

LANGDON JD AND PARTRIDGE M. (1992). Expression of the tumour suppressor gene p53 in oral cancer. *Br. J. Oral Maxillofacial Surg.*, **30**, 214–220.

LEVINE AJ, PERRY ME, CHANG A, SILVER A, DITTMER D, WU M AND WELSH D. (1994). The 1993 Walter Hubert lecture: the role of the p53 tumour-suppressor gene in tumorigenesis. *Br. J. Cancer*, **69**, 409–416.

LEWIN B. (1990). Nuclear splicing junctions may be interchangeable. In: *Genes IV*, pp. 596–598. Oxford University Press: Oxford.

McCORMICK D AND HALL PA. (1992). The complexities of proliferating cell nuclear antigen. *Histopathology*, **21**, 591–594.

MACK DH, VARTIKAR J, PIPAS JM AND LAIMINIS LA. (1993). Specific repression of TATA-mediated but not initiator-mediated transcription by wild-type p53. *Nature*, **363**, 281–283.

MARCHETTI A, BUTTITTA F, PELLEGRINI S, CAMPANI D, DIELLA F, CECCHETTI D, CALLAHAN R AND BISTOCCHI M. (1993). p53 mutations and histological type of invasive breast carcinoma. *Cancer Res.*, **53**, 4665–4669.

MERCER WE, SHIELDS MT, LIN D, APPELLA E AND ULLRICH SJ. (1991). Growth suppression induced by wild-type p53 protein is accompanied by selective down-regulation of proliferating-cell nuclear antigen expression. *Proc. Natl Acad. Sci. USA*, **88**, 1958–1962.

MICHAUD J, BRODY LC, STEEL G, FONTAINE G, MARTIN LS, VALLE D AND MITCHELL G. (1992). Strand-separating conformational polymorphism analysis: Efficacy of detection of point mutations in the human ornithine δ-aminotransferase gene. *Genomics*, **13**, 389–394.

NYLANDER K, ANNEROTH G, GUSTAFSSON H, ROOS G, STENLING R AND ZACKRISSON B. (1994). Cell kinetics of head and neck squamous cell carcinomas, prognostic implications. *Acta Oncol.*, **33**, 23–28.

NYLANDER K, STENLING R, GUSTAFSSON H, ZACKRISSON B AND ROOS G. (1995). p53 expression and cell proliferation in squamous cell carcinomas of the head and neck region. *Cancer* (in press).

OLINER JD, PIETENPOL JA, THIAGALINGAM S, GYURIS J, KINZLER KW AND VOGELSTEIN B. (1993). Oncoprotein MDM2 conceals the activation domain of tumour suppressor p53. *Nature*, **362**, 857–860.

ORITA M, SUZUKI Y, SEKIYA T AND HAYASHI K. (1989). Rapid and sensitive detection of point mutations and DNA polymorphisms using the polymerase chain reaction. *Genomics*, **5**, 874–879.

PERWEZ HUSSAIN S, AGUILAR F AND CERUTTI P. (1994). Mutagenesis of codon 248 of the human p53 tumour suppressor gene by N-ethyl-N-nitrosurea. *Oncogene*, **9**, 13–18.

RUBIO MP, VON DEIMLING A, YANDELL DW, WIESTLER OD, GUSELLA JF AND LOUIS DN. (1993). Accumulation of wild type p53 protein in human astrocytomas. *Cancer Res.*, **53**, 3465–3467.

SHIBATA DK. (1992). The polymerase chain reaction and the molecular genetic analysis of tissue biopsies. In *Diagnostic Molecular Pathology, a Practical Approach, Vol. II*, Herrington CS and McGee JO'D (eds) pp. 85–111. Oxford University Press: Oxford.

SOMERS KD, MERRICK MA, LOPEZ ME, INCOGNITO LS, SCHECHTER GL AND CASEY G. (1992). Frequent p53 mutations in head and neck cancer. *Cancer Res.*, **52**, 5997–6000.

THORLACIUS S, BORRESEN AL AND EYFJÖRD JE. (1993). Somatic p53 mutations in human breast carcinomas in an Icelandic population: a prognostic factor. *Cancer Res.*, **53**, 1637–1641.

VOJTESEK B, BARTEK J, MIDGLEY CA AND LANE DP. (1992). An immunochemical analysis of the human nuclear phosphoprotein p53. New monoclonal antibodies and epitope mapping using recombinant p53. *J. Immunol. Methods*, **151**, 237–244.

WAGATA T, SHIBAGAKI I, IMAMURA M, SHIMADA Y, TOGUCHIDA J, YANDELL DW, IKENAGA M, TOBE T AND ISHIZAKI K. (1993). Loss of 17p, mutation of the p53 gene, and overexpression of p53 protein in esophageal squamous cell carcinomas. *Cancer Res.*, **53**, 846–850.

WU JK, YE Z AND DARRAS BT. (1993). Sensitivity of single-strand conformation polymorphism (SSCP) analysis in detecting p53 point mutations in tumours with mixed cell populations. *Am. J. Hum. Genet.*, **52**, 1273–1275.

WYNFORD-THOMAS D. (1992). p53 in tumour pathology: can we trust immunocytochemistry? *J. Pathol.*, **166**, 329–330.